# Associations of combined work schedules and atypical working hours with mental health among South Korean police officers: a cross-sectional study

**DOI:** 10.1186/s12889-026-26706-9

**Published:** 2026-02-21

**Authors:** Jungwon Jang, Joungsue Kim, Youngjin Choi, Jeehee Min, Inah Kim

**Affiliations:** 1 Department of Occupational and Environmental Medicine, College of Medicine, Hanyang University, 222 Wangsimni‐ro, Seongdong‐gu, Seoul, 04763 Republic of Korea; 2 Department of Public Health, General Graduate School, Hanyang University, Seoul, Republic of Korea; 3 Department of Occupational and Environmental Medicine, Hanyang University Seoul Hospital, Seoul, Republic of Korea

**Keywords:** First responder, Shift work, Work schedule, Sleep, Mental health, Occupational health, Cross-sectional studies

## Abstract

**Background:**

Atypical working hours, including long working hours and weekend work, are established psychosocial risk factors for poor mental health and suicide in the general working population. Despite South Korea’s demanding working environment—characterized by extended hours—the mental health effects of combined atypical working hours and work schedules remain poorly understood, particularly in high-risk occupations such as policing. This study compared mental health outcomes among police officers according to combinations of work schedules and atypical working hours.

**Methods:**

This cross-sectional study analyzed data from 1,087 police officers collected in 2022 as part of the Mental Health Cohort of Police Officers in Korea. Combined work patterns were defined by work schedules (rotating shift work vs. fixed-day work) and atypical working hours: long working hours (> 52 h/week) and weekend work (≥ 4 h on weekends). Associations with mental health outcomes were examined using Poisson regression to estimate prevalence ratios and 95% confidence intervals (CIs). Analyses were stratified by sex for work schedule comparisons and pooled with sex adjustment for combined patterns to ensure statistical power.

**Results:**

Rotating shift work was associated with a higher prevalence of insomnia than fixed-day work, with more than a twofold increase among female officers (multivariable-adjusted prevalence ratio [aPR] = 2.38, 95% CI = 1.11–5.11). In pooled analyses, rotating shift work with or without long working hours was associated with insomnia compared with fixed-day work without long working hours or weekend work. Notably, fixed-day work combined with both long working hours and weekend work exhibited the highest aPRs for insomnia (aPR = 2.76, 95% CI = 1.23–6.16) and anxiety (aPR = 2.50, 95% CI = 1.27–4.89), and was significantly associated with depression (aPR = 1.61, 95% CI = 1.04–2.52).

**Conclusions:**

Rotating shift work was associated with insomnia, whereas the combination of long working hours and weekend work within fixed-day work showed the strongest associations with insomnia, depression, and anxiety. These results suggest that circadian variability primarily affects sleep among shift workers, whereas psychosocial variability (e.g., mandatory or unpredictable overtime) may contribute to insomnia, depression, and anxiety concurrently among fixed-day workers. Organizational interventions to limit mandatory overtime and reduce schedule unpredictability are urgently needed to protect police officers’ mental health.

**Supplementary Information:**

The online version contains supplementary material available at 10.1186/s12889-026-26706-9.

## Background

In South Korea, suicide rates among police officers have been increasing [1]. A well-established primary risk factor is shift work, which is an essential component of policing. Shift work, particularly night shift work, is associated with a wide range of adverse outcomes, including sleep disturbances [2–5], various physical and mental health problems, suicidal ideation [6–13], and increased social stress and job dissatisfaction among police officers [14]. However, several studies have suggested that maintaining the same shift pattern over time—regardless of the specific time of day—may mitigate these adverse effects, indicating that the consistency of work schedules itself plays a critical role in mental health [15, 16].

Extended working hours are a defining characteristic of Korea’s working environment and are expected to be another major risk factor for police officers’ mental health [17, 18]. For the general workforce, long working hours and weekend work are associated with poorer mental health and higher mortality from overwork-related diseases, including suicide [19–26]. A study among South Korean non-shift workers showed that the risk of depression and anxiety was highest when long working hours were combined with variability in working hours [19]. In South Korea, the potential influence of this distinctive working environment on police officers’ psychological distress and suicide has been repeatedly reported [1, 27]. However, empirical research specifically investigating this association remains scarce compared with that on rotating shift work.

In this context, the work patterns of South Korean police officers consist of various combinations of work schedules and atypical working hours. Typically, work schedules are classified as either rotating shift work or fixed-day work, and each schedule type may or may not include forms of atypical working hours, such as long working hours or weekend work. To our knowledge, no studies have investigated the associations of these combined work patterns with mental health among police officers. Therefore, this study aims to compare the prevalence of mental health outcomes across distinct combinations of work schedules and atypical working hours among South Korean police officers.

## Methods

### Study population and data source

**Fig. 1:**
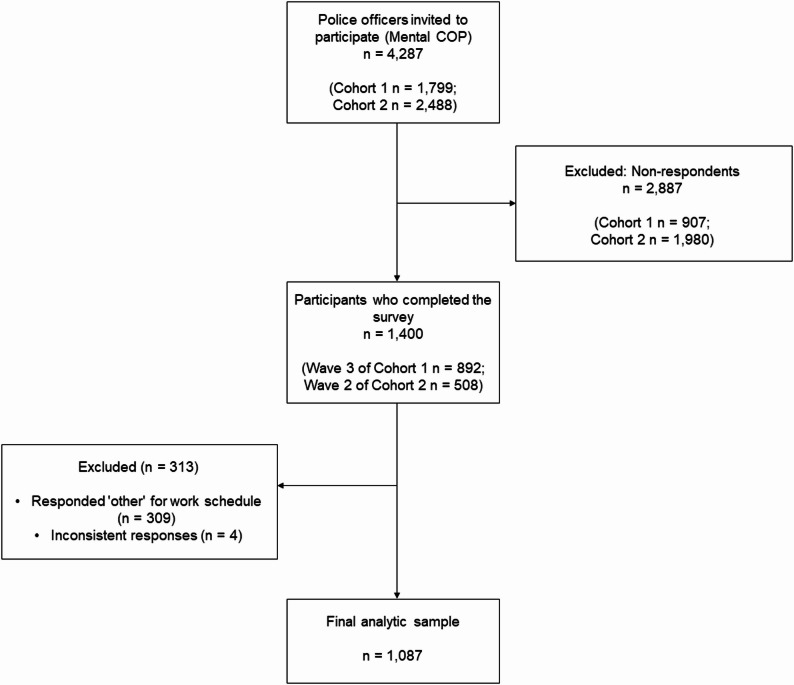
Flow diagram of study participants

The Mental Health Cohort of Police Officers in Korea (Mental COP) is a prospective cohort study designed to monitor the mental health of newly recruited police officers over time. Details of the cohort profile have been reported previously [28]. For this cross-sectional analysis, we used data from Wave 3 of Cohort 1 (five years after enrollment in 2017) and Wave 2 of Cohort 2 (three years after enrollment in 2019), both of which were collected between September and December 2022. A flow diagram illustrating the participant selection process is provided in Fig. 1. A total of 4,287 police officers were invited to participate in the follow-up survey (1,799 from Cohort 1 and 2,488 from Cohort 2). Of these, 2,887 were non-respondents (907 and 1,980 in each cohort, respectively), resulting in 1,400 participants who completed the survey.

All participants provided written informed consent prior to participation. This study was approved by the Institutional Review Board of Hanyang University, Seoul, Republic of Korea (HYI-15-213-18, HYUIRB-2019-06-004-11, and HYUIRB-2023-04-001-7).

### Combinations of work schedules and atypical working hours

As official records of police officers’ work schedules and working hours are not publicly available in Korea, we used self-reported data on work patterns from the month of the survey.

We defined work schedules and atypical working hours as the two main components of combined work patterns. Work schedules were classified as either fixed-day work, defined as a 9:00–18:00 schedule five days per week, or rotating shift work, defined as rotating shifts that included night shifts. Additionally, two types of atypical working hours were identified: long working hours, defined as working more than 52 h per week, based on the Korean legal standard and previous studies [18, 19, 21]; and weekend work, defined as working four or more hours on a weekend at least once per month.

**Fig. 2:**
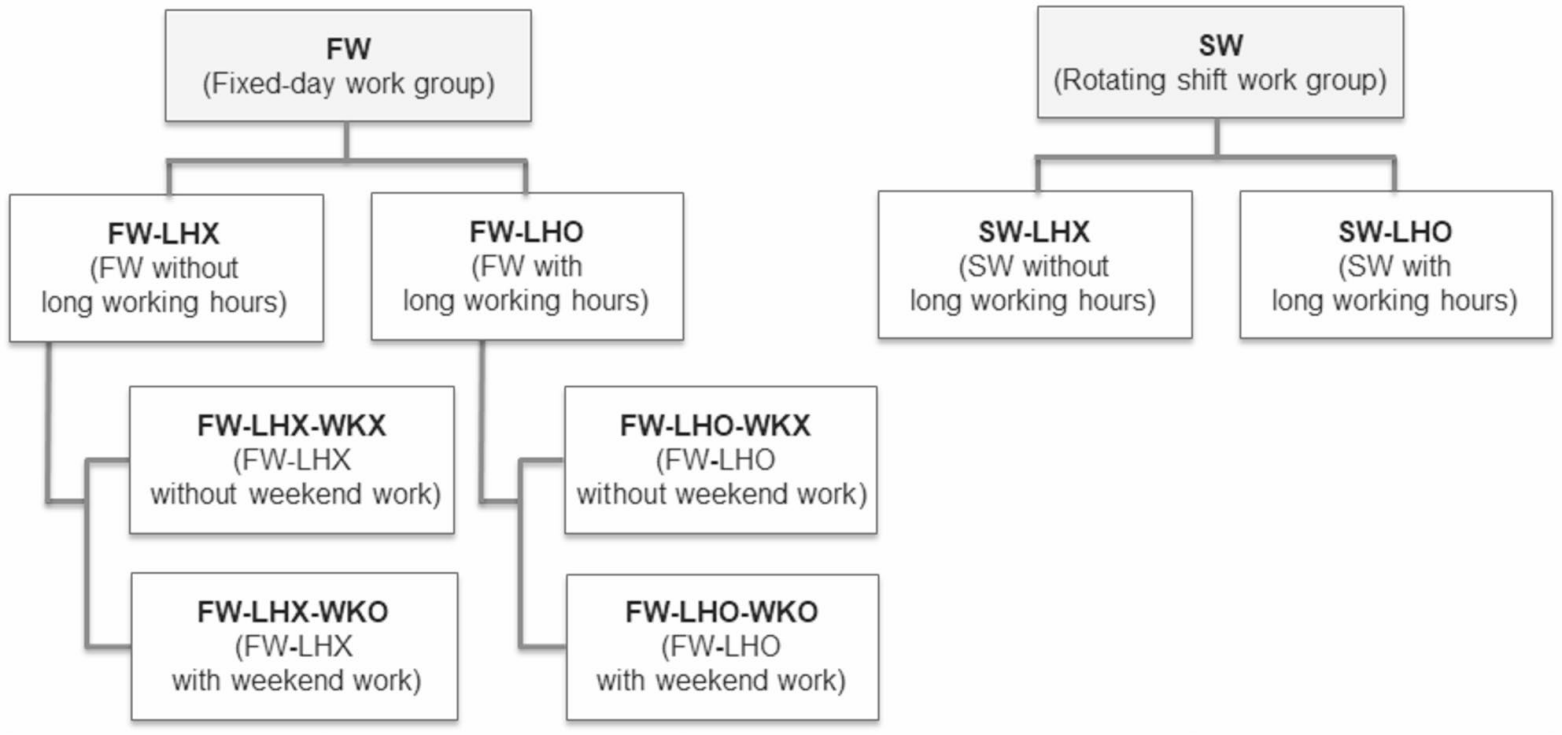
Classification of combined work schedules and atypical working hours

For the analysis, we constructed the combinations of work schedules and atypical working hours in the following sequence (Fig. 2). First, participants were stratified by work schedules into the fixed-day work group (FW) and the rotating shift work group (SW). Second, each of these categories was further divided based on long working hours (without [LHX] vs. with [LHO]). Finally, considering that weekend work is an intrinsic characteristic of rotating shift work in policing, the classification based on weekend work (without [WKX] vs. with [WKO]) was applied only to the FW. Consequently, a total of six combinations were established for the final analysis: two within the SW (SW-LHX and SW-LHO) and four within the FW (FW-LHX-WKX, FW-LHX-WKO, FW-LHO-WKX, and FW-LHO-WKO).

### Self-reported mental health assessments

Mental health outcomes were measured for insomnia, depression, and anxiety using standardized assessment instruments validated for Korean populations. (1) Insomnia: assessed using the Korean version of the Insomnia Severity Index (ISI-K) [29]; score range: 0–28, with moderate insomnia defined as a score of ≥ 15. (2) Depression: assessed using the Korean version of the Patient Health Questionnaire-9 (PHQ-9) [30]; score range: 0–27, with mild depression defined as a score of ≥ 5. (3) Anxiety: assessed using the Korean version of the Generalized Anxiety Disorder 7-item scale (GAD-7) [31]; score range: 0–21, with mild anxiety defined as a score of ≥ 5.

### Covariates

Sociodemographic covariates were collected through self-report and included sex, age group (< 30 or ≥ 30 years), marital status (married or not married), monthly household income (low, lower-middle, upper-middle, or high), education level (≤ high school, college < 4 years, or college ≥ 4 years), smoking status (never, past, or current), alcohol consumption (≤ 1 time per week or > 1 time per week), and rank (patrol officer, senior patrol officer, or sergeant).

### Statistical analysis

The chi-square test or Fisher’s exact test was used to compare sociodemographic characteristics and mental health outcomes across combinations of work schedules and atypical working hours, as well as to compare baseline characteristics between the analytic sample and excluded participants to assess potential selection bias. As odds ratios derived from logistic regression in cross-sectional studies can overestimate prevalence, we used Poisson regression with robust variance to analyze the data [32, 33]. Mental health outcomes were compared using prevalence rates, prevalence ratios (PRs), and their corresponding 95% confidence intervals (CIs). All PR analyses were performed using both age-adjusted and multivariable-adjusted models. The multivariable-adjusted prevalence ratios (aPRs) were estimated by adjusting for covariates selected as potential confounders based on previous literature on police officers’ mental health, namely age group, marital status, monthly household income, education level, smoking status, alcohol consumption, and rank. Analyses were stratified by sex; however, if no significant interaction was observed, sex was included as a covariate in the models. Additionally, three types of sensitivity analyses were performed. First, to account for potential differences between Cohort 1 and Cohort 2, cohort membership was included as a covariate in the model. Second, to assess robustness against the cutoff values used for binary classification, linear regression models were utilized with continuous scores for the ISI-K, PHQ-9, and GAD-7. Finally, analyses were repeated including the four participants initially excluded due to inconsistent responses. All statistical analyses were performed using SAS software (version 9.4; SAS Institute Inc., Cary, North Carolina, USA).

## Results

Of the 1,400 police officers surveyed, we excluded 309 who selected ‘other’ as their work schedule and four with inconsistent responses (e.g., reporting rotating night shifts but indicating no weekend or night work). The final analytic sample thus consisted of 1,087 police officers.

For the attrition analysis to assess potential selection bias (see Supplementary Table 1), we compared the analytic sample (*n* = 1,075; successfully linked to baseline [Wave 1] records) with the excluded participants. The excluded group comprised non-respondents, participants excluded based on study criteria, and 12 individuals (all from Cohort 1) from the final analytic sample whose identification markers could not be linked to baseline records. No significant differences were observed in age group, sex, insomnia, or anxiety; comparisons involving anxiety were restricted to Cohort 2 because the GAD-7 was not assessed in Wave 1 of Cohort 1. However, the analytic sample exhibited significantly higher proportions of depression, university graduates (≥ 4 years of college), and low-income households compared with excluded participants.

Of this final sample, 818 were male, and 269 were female (Table 1). Males were predominantly represented in the SW combinations (SW-LHX: 43.5%; SW-LHO: 15.6%), whereas females were concentrated in FW-LHX (55.8%).

**Table 1:** Sociodemographic characteristics of Police officers by combinations of work schedules and long working hours

		FW-LHX		FW-LHO		SW-LHX		SW-LHO		
		*N*	(%)	*N*	(%)	*N*	(%)	*N*	(%)	*p*-value
Male (*n* = 818)		242	(29.6)	92	(11.2)	356	(43.5)	128	(15.6)	
Age group (years)	< 30	77	(31.8)	29	(31.5)	71	(19.9)	34	(26.6)	0.0057
	≥ 30	165	(68.2)	63	(68.5)	285	(80.1)	94	(73.4)	
Marital status	Not married	154	(63.6)	54	(58.7)	194	(54.5)	70	(54.7)	0.1383
	Married	88	(36.4)	38	(41.3)	162	(45.5)	58	(45.3)	
Monthly household income	Low	8	(3.3)	1	(1.1)	2	(0.6)	0	(0.0)	< 0.0001
	Lower-middle	134	(55.4)	49	(53.3)	156	(43.8)	52	(40.6)	
	Upper-middle	50	(20.7)	20	(21.7)	105	(29.5)	47	(36.7)	
	High	50	(20.7)	22	(23.9)	93	(26.1)	29	(22.7)	
Education level	≤ High school	22	(9.1)	3	(3.3)	34	(9.6)	5	(3.9)	0.0849
	College < 4 years	107	(44.2)	34	(37.0)	137	(38.5)	51	(39.8)	
	College ≥ 4 years	113	(46.7)	55	(59.8)	185	(52.0)	72	(56.3)	
Smoking status	Never	101	(41.7)	43	(46.7)	156	(43.8)	47	(36.7)	0.5149
	Past	73	(30.2)	21	(22.8)	104	(29.2)	36	(28.1)	
	Current	68	(28.1)	28	(30.4)	96	(27.0)	45	(35.2)	
Alcohol consumption	≤ 1/per week	188	(77.7)	71	(77.2)	265	(74.4)	100	(78.1)	0.7498
	> 1/per week	54	(22.3)	21	(22.8)	91	(25.6)	28	(21.9)	
Rank	Patrol officer	26	(10.7)	10	(10.9)	64	(18.0)	16	(12.5)	0.0164
	Senior patrol	175	(72.3)	64	(69.6)	258	(72.5)	95	(74.2)	
	Sergeant	41	(16.9)	18	(19.6)	34	(9.6)	17	(13.3)	
Female (*n* = 269)		150	(55.8)	32	(11.9)	64	(23.8)	23	(8.6)	
Age group (years)	< 30	62	(41.3)	19	(59.4)	31	(48.4)	12	(52.2)	0.2484
	≥ 30	88	(58.7)	13	(40.6)	33	(51.6)	11	(47.8)	
Marital status	Not married	86	(57.3)	21	(65.6)	47	(73.4)	16	(69.6)	0.1336
	Married	64	(42.7)	11	(34.4)	17	(26.6)	7	(30.4)	
Monthly household income	Low	22	(14.7)	4	(12.5)	0	(0.0)	0	(0.0)	0.0002
	Lower-middle	56	(37.3)	4	(12.5)	26	(40.6)	7	(30.4)	
	Upper-middle	31	(20.7)	11	(34.4)	8	(12.5)	9	(39.1)	
	High	41	(27.3)	13	(40.6)	30	(46.9)	7	(30.4)	
Education level	≤ High school	7	(4.7)	2	(6.3)	2	(3.1)	4	(17.4)	< 0.0001
	College < 4 years	38	(25.3)	12	(37.5)	13	(20.3)	8	(34.8)	
	College ≥ 4 years	105	(70.0)	18	(56.3)	49	(76.6)	11	(47.8)	
Smoking status	Never	124	(82.7)	23	(71.9)	59	(92.2)	18	(78.3)	< 0.0001
	Past	22	(14.7)	5	(15.6)	3	(4.7)	3	(13.0)	
	Current	4	(2.7)	4	(12.5)	2	(3.1)	2	(8.7)	
Alcohol consumption	≤ 1/per week	120	(80.0)	25	(78.1)	54	(84.4)	12	(52.2)	0.0121
	> 1/per week	30	(20.0)	7	(21.9)	10	(15.6)	11	(47.8)	
Rank	Patrol officer	24	(16.0)	5	(15.6)	13	(20.3)	1	(4.4)	< 0.0001
	Senior patrol	113	(75.3)	23	(71.9)	45	(70.3)	19	(82.6)	
	Sergeant	13	(8.7)	4	(12.5)	6	(9.4)	3	(13.0)	

*FW* Fixed-day work group, *SW* Rotating shift work group, *LHX* without long working hours, *LHO* with long working hours

We compared the PRs of mental health outcomes between the FW and the SW stratified by sex, as a significant interaction between work schedule and sex was observed for depression (P for interaction = 0.0324) (Table 2). In both male and female officers, the SW was associated with a higher prevalence of insomnia than the FW. Notably, female officers showed more than twice the prevalence of insomnia in the SW compared to the FW (aPR = 2.38, 95% CI = 1.11–5.11).

**Table 2:** Prevalence ratios of mental health outcomes by work schedules, stratified by sex

	FW	SW	
Male			
Insomnia			
Prevalence, %	8.7	14.7	
Model 1 PR (95% CI)*	1.00	**1.66**	**(1.07–2.56)**
Model 2 PR (95% CI)†	1.00	**1.68**	**(1.08–2.62)**
Depression			
Prevalence, %	27.3	23.4	
Model 1 PR (95% CI)*	1.00	0.85	(0.64–1.12)
Model 2 PR (95% CI)†	1.00	0.85	(0.64–1.13)
Anxiety			
Prevalence, %	10.5	8.1	
Model 1 PR (95% CI)*	1.00	0.73	(0.46–1.16)
Model 2 PR (95% CI)†	1.00	0.73	(0.46–1.17)
Female			
Insomnia			
Prevalence, %	9.3	19.5	
Model 1 PR (95% CI)*	1.00	**2.04**	**(1.04–4.01)**
Model 2 PR (95% CI)†	1.00	**2.38**	**(1.11–5.11)**
Depression			
Prevalence, %	36.3	47.1	
Model 1 PR (95% CI)*	1.00	1.30	(0.88–1.93)
Model 2 PR (95% CI)†	1.00	1.50	(0.97–2.31)
Anxiety			
Prevalence, %	19.2	20.7	
Model 1 PR (95% CI)*	1.00	1.09	(0.62–1.93)
Model 2 PR (95% CI)†	1.00	1.16	(0.62–2.16)

*FW* Fixed-day work group, *SW* Rotating shift work group

*Prevalence ratio and 95% confidence interval adjusted for age group

†Multivariable-adjusted prevalence ratio (aPR) and 95% confidence interval adjusted for age group, marital status, monthly household income, education level, smoking status, alcohol consumption, and rank ; bold values indicate statistical significance (p<0.05).

Table 3 presents the PRs of mental health outcomes according to the six combinations of work schedules and atypical working hours. Since no significant interactions were observed between sex and these combinations for any mental health outcomes, we presented the results of the pooled analysis, adjusting for sex. FW-LHO-WKO exhibited significantly higher aPRs for all three mental health outcomes compared to those for FW-LHX-WKX. Specifically, the aPRs of insomnia and anxiety were more than twofold higher in FW-LHO-WKO (aPR = 2.76, 95% CI = 1.23–6.16; aPR = 2.50, 95% CI = 1.27–4.89, respectively), and the aPR of depression was also significantly higher (aPR = 1.61, 95% CI = 1.04–2.52). Both SW-LHX and SW-LHO showed significantly higher aPRs for insomnia (aPR = 2.76, 95% CI = 1.45–5.24; aPR = 3.02, 95% CI = 1.48–6.17, respectively) compared to FW-LHX-WKX, while their associations with depression and anxiety were not statistically significant. Sensitivity analyses assessing robustness to the cutoff values used for binary classification (see Supplementary Table 2) showed that results from linear regression models using continuous scores of mental health outcomes were generally consistent with the findings presented in Table 3.

**Table 3:** Pooled prevalence ratios of mental health outcomes according to the six combinations of work schedules and atypical working hours

	FW-LHX -WKX	FW-LHX-WKO	FW-LHO-WKX	FW-LHO-WKO	SW-LHX	SW-LHO
n (%)	197 (18.1%)	195 (17.9%)	36 (3.3%)	88 (8.1%)	420 (38.6%)	151 (13.9%)
Insomnia						
Prevalence, %	6.1	10.8	0.0	14.8	15.0	16.6
Model 1 PR (95% CI)*	1.00	1.81 (0.89–3.69)	N/A	**2.52** **(1.15–5.56)**	**2.63** **(1.40–4.94)**	**2.90** **(1.44–5.85)**
Model 2 PR (95% CI)†	1.00	1.78 (0.87–3.63)	N/A	**2.76** **(1.23–6.16)**	**2.76** **(1.45–5.24)**	**3.02** **(1.48–6.17)**
Depression						
Prevalence, %	27.4	30.8	27.8	37.5	25.7	30.5
Model 1 PR (95% CI)*	1.00	1.20 (0.83–1.74)	1.14 (0.58–2.24)	1.52 (0.98–2.35)	1.09 (0.78–1.53)	1.30 (0.87–1.94)
Model 2 PR (95% CI)†	1.00	1.19 (0.82–1.73)	1.10 (0.56–2.19)	**1.61** **(1.04–2.52)**	1.14 (0.81–1.60)	1.33 (0.89–2.00)
Anxiety						
Prevalence, %	9.6	14.4	16.7	19.3	9.3	11.9
Model 1 PR (95% CI)*	1.00	1.72 (0.96–3.09)	2.22 (0.88–5.60)	**2.48** **(1.28–4.80)**	1.26 (0.72–2.22)	1.66 (0.86–3.22)
Model 2 PR (95% CI)†	1.00	1.69 (0.94–3.04)	2.10 (0.82–5.35)	**2.50** **(1.27–4.89)**	1.30 (0.73–2.31)	1.57 (0.80–3.06)

*FW* Fixed-day work group, *SW* Rotating shift work group, *LHX* without long working hours, *LHO* with long working hours, *WKX* without weekend work,* WKO* with weekend work

*Prevalence ratio and 95% confidence interval adjusted for age group and sex

†Multivariable-adjusted prevalence ratio (aPR) and 95% confidence interval adjusted for age group, sex, marital status, monthly household income, education level, smoking status, alcohol consumption, and rank ; bold values indicate statistical significance (p<0.05).

To investigate sex-specific patterns in the associations between combinations of work schedules and atypical working hours, analyses of FW combinations were stratified by sex (see Supplementary Table 3). FW-LHO-WKO was significantly associated with depression and anxiety among male officers, whereas among female officers it was significantly associated only with insomnia.

Finally, sensitivity analyses adjusting for cohort membership and including the four participants excluded due to inconsistent responses yielded results consistent with the main analysis (data not shown).

## Discussion

This study revealed that the mental health problems associated with police officers’ work schedules are not confined to rotating shift work but vary significantly depending on the combinations of atypical working hours. Notably, the most critical finding was that the combination of long working hours and weekend work among officers with fixed-day work schedules was associated with substantially elevated prevalence of insomnia, depression, and anxiety. This suggests that, even among police officers within fixed-day work arrangements, schedule unpredictability and excessive workload serve as significant risk factors for adverse mental health outcomes.

Our finding that the prevalence of insomnia was higher among both male and female officers working rotating shifts than among those on fixed-day schedules is consistent with previous studies that compared police officers working rotating shifts with those working non-rotating shifts [14, 15]. However, it remains a matter of debate whether sleep problems are driven primarily by the rotation itself or by night work. Some studies comparing different work shifts have reported that night shifts, in particular, are associated with sleep problems due to physiological factors and exposure to more serious stress events [10, 13, 34, 35]. Conversely, research focusing on the work pattern itself suggests that circadian variability is the key issue. For example, transitioning from day work to night work can induce irregular sleep–wake and mealtime behaviors [36]. Furthermore, maintaining a long-term consistent schedule, even when it includes night work, has been found to improve sleep quality and psychological well-being [15, 16]. These findings collectively suggest that the adverse effects on sleep may stem more from circadian variability than from the specific time of day, underscoring the need for further investigation.

Despite many police officers in South Korea working long hours or weekends, extended working hours have received less attention than rotating shift work, and even fewer studies have examined the mental health consequences of fixed-day work. Notably, in contrast to analyses comparing only rotating shift work and fixed-day work, analyses of specific combinations of long working hours and weekend work showed that officers in FW-LHO-WKO—who worked long hours and weekends—had a markedly higher prevalence of insomnia, depression, and anxiety than those in FW-LHX-WKX, who had neither long working hours nor weekend work. This finding may reflect heavy workloads, urgent tasks, and increased exposure to unexpected and mandatory overtime, suggesting that the demanding aspects of extended working hours—common in the Korean working environment—are also present among fixed-day work officers [1, 27]. Previous studies have reported that long working hours and greater variability or irregularity in work patterns are linked to adverse psychosocial outcomes among both police officers and the general workforce [12, 15, 19–25, 37]. In particular, working 49 h or more per week was significantly associated with mental illness in police officers [12], and discrepancies between employees’ actual and desired working hours have been associated with depression among Korean workers [25]. In Japan, weekend work has been shown to have nearly twice the negative impact on mental health compared with weekday overtime [22]. Additionally, instability and unpredictability in working hours are strongly associated with psychological distress, including poor sleep quality, highlighting these factors as critical determinants for intervention in occupational mental health [38]. Furthermore, the association of long working hours and weekend work with mental health may vary significantly depending on their voluntariness and mandatory status. Although our study did not assess the voluntariness of overtime, shift-working police officers in Korea can voluntarily sign up for additional work during the day, at night, or during off-duty hours to compensate for departmental staffing shortages. Prior research has distinguished between types of overtime, suggesting that mandatory overtime, rather than voluntary overtime, may negatively impact mental health [15].

However, unlike FW-LHO-WKO, the SW combinations (SW-LHX and SW-LHO) showed a high prevalence of insomnia but not depression, which is inconsistent with previous studies identifying insomnia as a strong predictor of depression [39]. This discrepancy might be attributed to unmeasured factors in this study, such as the voluntariness of overtime, actual sleep duration, or the frequency of weekend work. Indeed, a previous cross-sectional study observed that the association between long working hours and depression was not significant when individuals maintained a sleep duration of at least six hours or reported subjective sleep sufficiency [40]. To clarify these pathways, future research should expand on sleep duration and weekend work frequency to further examine dose-response relationships with mental health outcomes.

Some of our findings suggest sex-differentiated patterns. In the analysis of the FW combinations (see Supplementary Table 3), male officers in FW-LHO-WKO showed significant associations with depression and anxiety, whereas female officers with the same combination exhibited a statistically significant association only with insomnia. These results may reflect the influence of unmeasured factors, such as the gendered occupational context in Korea and family care responsibilities. A previous study suggested that the lack of a significant association between work-related stressors and suicidal ideation among middle-aged Korean female workers could be attributed to the stronger influence of non-work stressors, such as family care [41]. However, the failure to reach statistical significance for depression and anxiety among female officers in this combination may stem from limited statistical power due to the study’s small sample size, rather than from the absence of association.

Collectively, these results suggest that variability in working hours and mandatory overtime may represent a core mechanism underlying mental health problems. Specifically, our findings suggest two differentiated pathways: circadian variability within rotating shift work appears to primarily relate to sleep problems, whereas psychosocial variability—such as mandatory overtime and unpredictable schedules—may be more directly associated with sleep as well as depression and anxiety. Notably, while no significant associations were observed in other FW combinations exposed to either long working hours alone (FW-LHO-WKX) or weekend work alone (FW-LHX-WKO), significantly higher prevalence rates across all mental health outcomes were observed only in FW-LHO-WKO, where both factors were combined. This suggests that the cumulative burden of atypical working hours is associated with a broader range of mental health outcomes. This finding is consistent with previous studies suggesting that long working hours and weekend work may be adversely linked to mental health through mechanisms involving work–life imbalance, chronic stress, and insufficient recovery [19, 22–24].

This study had several limitations. First, it was based on cross-sectional data drawn from the Mental COP cohort, which precludes causal inference between work patterns and mental health problems. Although the data collection was conducted in late 2022, after the COVID-19 pandemic, changes in administrative demands or staffing shortages may have influenced the mental health of police officers compared with the pandemic period; however, the cross-sectional design could not capture such contextual variations. Second, the findings are limited in their representativeness and generalizability. The study sample consisted of newly recruited police officers with three to five years of service drawn from the Mental COP cohort. This specific sample composition implies that the sample may not fully represent the entire South Korean police population. Moreover, the high non-response rate suggests that the healthy worker effect cannot be ruled out. However, comparison of baseline characteristics revealed that the analytic sample had a higher prevalence of depression than the excluded participants, suggesting that potential bias from the healthy worker effect may not necessarily lead to underestimation of the results. In addition, the findings regarding the high-risk FW-LHO-WKO may reflect conditions specific to Korea’s work environment, characterized by long working hours and overwork, thereby limiting generalizability to police populations in countries with stricter working-time regulations. To address these limitations, we are conducting an ongoing long-term follow-up of existing participants, continuously enrolling new police officers into the cohort to improve representativeness, and developing a dedicated mobile application for police health management to enhance participation and retention rates in future waves. Third, all variables—including work schedules, working hours, and mental health—were based on self-reported measures. However, we found a high degree of internal consistency in responses to similar questions. Only four participants with inconsistent answers (e.g., reporting rotating night shifts but no weekend or night work) were excluded. Among the remaining respondents, 100% of those who reported shift work also indicated working at least one night and/or weekend. Korean public officials may officially record fewer overtime hours than those actually worked because the maximum number of payable overtime hours is limited by regulations on allowances for public officials [42]. Therefore, self-reporting may provide a more accurate reflection of total working hours. Previous research has also demonstrated good agreement between self-reported mental health data and medical records [43]. Finally, a broader range of personal and occupational factors—such as police duties, voluntary overtime, sleep duration, frequency of weekend work, sick leave, absenteeism, discrepancies between actual and desired working hours, family composition, the presence of children, and time spent outside work—were not considered.

Nevertheless, this is the first study to investigate the associations between diverse combinations of work schedules and atypical working hours and the prevalence of mental health outcomes among police officers. Such extreme and complex combinations may have been overlooked in prior research because they are rare in other countries. South Korea ranks among the top OECD countries for long working hours [17, 18], and overtime and unpredictable weekend work are prevalent even among police officers, who are civil servants. The conventional dichotomy of rotating shift work versus fixed-day work is insufficient to capture the complex risks present in high-demand, long-hour work environments such as the Korean police force. Comprehensive longitudinal research is essential to clarify causal relationships and inform evidence-based policy development. Future studies should consider integrating self-reported data with objective administrative records (e.g., human resource data) and incorporating relevant occupational and personal factors.

## Conclusions

In this cross-sectional study of South Korean police officers, combinations of work schedules and atypical working hours were associated with diverse mental health outcomes. In particular, the combination of long working hours and weekend work within fixed-day work (FW-LHO-WKO) showed a substantially higher prevalence of insomnia, depression, and anxiety. These findings suggest that fixed-day work schedules do not necessarily protect mental well-being, and that irregular or unpredictable working patterns—especially those involving involuntary or mandatory overtime—may further contribute to psychological risks. These results underscore the need for targeted interventions to mitigate the psychosocial risks associated with long working hours and weekend work among police officers. Policies that ensure compliance with maximum working-hour regulations, prohibit excessive mandatory overtime, guarantee adequate rest periods, and design shift systems that minimize health risks may help protect the mental health of police officers and enhance the safety of the communities they serve [44].

## Supplementary Information

Supplementary Material 1.

## Data Availability

The datasets contain personal, identifiable, and sensitive information; therefore, public access is restricted in accordance with the regulations of the Personal Information Protection Commission of Korea. Data are available from the corresponding author, Prof. Inah Kim, upon reasonable request.

## Abbreviations

Mental COP: The Mental Health Cohort of Police Officers in Korea
FW: Fixed-day work group
SW: Rotating shift work group
LHX: Without long working hours
LHO: With long working hours
WKX: Without weekend work
WKO: With weekend work
ISI-K: Korean version of the Insomnia Severity Index
PHQ-9: Korean version of the Patient Health Questionnaire-9
GAD-7: Korean version of the Generalized Anxiety Disorder 7-item scale
PR: Prevalence ratio
CI: 95% confidence interval
