# Supplementary material for: Associations of combined work schedules and atypical working hours with mental health among South Korean police officers: a cross-sectional study
Source: BMC Public Health. 2026 Feb 21;26:1028. doi: 10.1186/s12889-026-26706-9 (PMC13032631; doi:10.1186/s12889-026-26706-9)
Supplement: Supplementary file 1 — Supplementary Material 1. [file 12889_2026_26706_MOESM1_ESM.docx]

Article title: Associations of combined work schedules and atypical working hours with mental health among South Korean police officers: A cross-sectional study

Journal name: BMC Public Health

Author names: Jungwon Jang, Joungsue Kim, Youngjin Choi, Jeehee Min, Inah Kim

Affiliation and e-mail address of the corresponding author:

Inah Kim, MD, MPH, PhD

Department of Occupational and Environmental Medicine, College of Medicine, Hanyang University, 222 Wangsimni‐ro, Seongdong‐gu, Seoul 04763, Republic of Korea

Telephone number: 82-2-2220-0665

Email address: inahkim@hanyang.ac.kr

Fax number: 82-2-2220-0699

**Supplementary Table 1**. Comparison of baseline (Wave 1) characteristics between analytic sample and excluded participants

|  |  | **Analytic sample**  **(n=1,075)** | | **Excluded participants**  **(n=3,212)** | |  |
| --- | --- | --- | --- | --- | --- | --- |
|  |  | **N** | **(%)** | **N** | **(%)** | **p-value** |
| Age group (years) | < 30 | 878 | (81.7) | 2,677 | (83.3) | 0.208 |
|  | ≥ 30 | 197 | (18.3) | 535 | (16.7) |  |
| Sex | Male | 811 | (75.4) | 2,455 | (76.4) | 0.5094 |
|  | Female | 264 | (24.6) | 757 | (23.6) |  |
| Marital status | Not married | 1,034 | (96.5) | 3,130 | (97.7) | 0.0333 |
|  | Married | 38 | (3.5) | 75 | (2.3) |  |
| Monthly household income | Low | 369 | (36.7) | 892 | (28.9) | <.0001 |
|  | Lower-middle | 58 | (5.8) | 239 | (7.8) |  |
|  | Upper-middle | 165 | (16.4) | 626 | (20.3) |  |
|  | High | 414 | (41.2) | 1,326 | (43.0) |  |
| Education level | ≤ High school | 321 | (30.1) | 650 | (20.3) | <.0001 |
|  | College < 4 years | 200 | (18.7) | 1,257 | (39.2) |  |
|  | College ≥ 4 years | 547 | (51.2) | 1,298 | (40.5) |  |
| Insomnia (ISI-K) | < 15 | 1,047 | (97.4) | 3,124 | (97.3) | 0.854 |
|  | ≥ 15 | 28 | (2.6) | 87 | (2.7) |  |
| Depression (PHQ-9) | < 5 | 969 | (90.3) | 3,024 | (94.2) | <.0001 |
|  | ≥ 5 | 104 | (9.7) | 185 | (5.8) |  |
| Anxiety (GAD-7)* | < 5 | 353 | (97.8) | 2,101 | (99.0) | 0.0578 |
|  | ≥ 5 | 8 | (2.2) | 22 | (1.0) |  |

Of the 1,087 participants in the final analytic sample, 1,075 were successfully linked to their baseline (Wave 1) records for the attrition analysis. The excluded participants comprised non-respondents, those who did not meet the study criteria, and 12 individuals from the final analytic sample whose identification markers could not be linked to baseline records.

*As the GAD-7 was not assessed in Wave 1 of Cohort 1, comparison was restricted to Cohort 2.

**Supplementary Table 2** Linear regression of continuous mental health scores (ISI-K, PHQ-9, and GAD-7) according to the six combinations of work schedules and atypical working hours

|  | **Insomnia** | **Depression** | **Anxiety** |
| --- | --- | --- | --- |
|  | β (95% CI) | β (95% CI) | β (95% CI) |
| FW-LHX-WKX | 0 (Ref) | 0 (Ref) | 0 (Ref) |
| FW-LHX-WKO | **1.20 (0.11–2.28)** | 0.42 (-0.27–1.11) | 0.41 (-0.12–0.93) |
| FW-LHO-WKX | -0.70 (-2.66–1.25) | -0.03 (-1.27–1.20) | 0.20 (-0.76–1.15) |
| FW-LHO-WKO | **1.68 (0.29–3.07)** | **1.07 (0.19–1.95)** | **0.75 (0.07–1.42)** |
| SW-LHX | **2.11 (1.15–3.07)** | 0.45 (-0.15–1.06) | 0.13 (-0.34–0.60) |
| SW-LHO | **2.20 (1.01–3.39)** | **0.77 (0.01–1.52)** | 0.28 (-0.30–0.85) |

FW, fixed-day work group; SW, rotating shift work group; LHX, without long working hours; LHO, with long working hours; WKX, without weekend work; WKO, with weekend work.

Linear regression models are adjusted for age group, sex, marital status, monthly household income, education level, smoking status, alcohol consumption, and rank.

**Supplementary Table 3** Prevalence ratios of mental health outcomes by combinations of long working hours and weekend work among fixed-day work groups

|  | **FW-LHX**  **-WKX** | **FW-LHX**  **-WKO** | | **FW-LHO**  **-WKX** | | **FW-LHO**  **-WKO** | |
| --- | --- | --- | --- | --- | --- | --- | --- |
| **Male, n (%)** | 111 (33.2) | 131 | (39.2) | 27 | (8.1) | 65 | (19.5) |
| Insomnia |  |  |  |  |  |  |  |
| Prevalence, % | 7.2 | 9.9 |  | 0.0 |  | 12.3 |  |
| Model 1 PR (95% CI)* | 1.00 | 1.36 | (0.56–3.29) | N/A |  | 1.70 | (0.64–4.54) |
| Model 2 PR (95% CI)† | 1.00 | 1.44 | (0.59–3.53) | N/A |  | 2.15 | (0.77–6.04) |
| Depression |  |  |  |  |  |  |  |
| Prevalence, % | 20.7 | 28.2 |  | 18.5 |  | 40.0 |  |
| Model 1 PR (95% CI)* | 1.00 | 1.37 | (0.82–2.31) | 0.90 | (0.34–2.37) | **1.93** | **(1.10–3.39)** |
| Model 2 PR (95% CI)† | 1.00 | 1.45 | (0.85–2.45) | 0.96 | (0.36–2.56) | **2.02** | **(1.14–3.60)** |
| Anxiety |  |  |  |  |  |  |  |
| Prevalence, % | 4.5 | 11.5 |  | 11.1 |  | 18.5 |  |
| Model 1 PR (95% CI)* | 1.00 | 2.58 | (0.94–7.11) | 2.50 | (0.60–10.48) | **4.12** | **(1.45–11.68)** |
| Model 2 PR (95% CI)† | 1.00 | 2.64 | (0.95–7.31) | 2.27 | (0.53–9.78) | **4.40** | **(1.52–12.76)** |
| **Female, n (%)** | 86 (47.3) | 64 | (35.2) | 9 | (4.9) | 23 | (12.6) |
| Insomnia |  |  |  |  |  |  |  |
| Prevalence, % | 4.7 | 12.5 |  | 0.0 |  | 21.7 |  |
| Model 1 PR (95% CI)* | 1.00 | 2.42 | (0.72–8.11) | N/A |  | **4.03** | **(1.07–15.25)** |
| Model 2 PR (95% CI)† | 1.00 | 2.11 | (0.60–7.42) | N/A |  | **4.95** | **(1.07–23.00)** |
| Depression |  |  |  |  |  |  |  |
| Prevalence, % | 36.1 | 35.9 |  | 55.6 |  | 30.4 |  |
| Model 1 PR (95% CI)* | 1.00 | 1.02 | (0.59–1.76) | 1.63 | (0.62–4.24) | 0.88 | (0.38–2.00) |
| Model 2 PR (95% CI)† | 1.00 | 0.99 | (0.56–1.76) | 1.15 | (0.42–3.18) | 0.88 | (0.36–2.13) |
| Anxiety |  |  |  |  |  |  |  |
| Prevalence, % | 16.3 | 20.3 |  | 33.3 |  | 21.7 |  |
| Model 1 PR (95% CI)* | 1.00 | 1.30 | (0.61–2.78) | 2.23 | (0.63–7.91) | 1.41 | (0.50–3.97) |
| Model 2 PR (95% CI)† | 1.00 | 1.21 | (0.54–2.67) | 1.89 | (0.50–7.19) | 1.13 | (0.37–3.48) |

FW, fixed-day work group; SW, rotating shift work group; LHX, without long working hours; LHO, with long working hours; WKX, without weekend work; WKO, with weekend work.

*Prevalence ratio and 95% confidence interval adjusted for age group.

†Multivariable-adjusted prevalence ratio (aPR) and 95% confidence interval adjusted for age group, marital status, monthly household income, education level, smoking status, alcohol consumption, and rank.
